# Physiological Status Drives Metabolic Rate in Mediterranean Geckos Infected with Pentastomes

**DOI:** 10.1371/journal.pone.0144477

**Published:** 2015-12-14

**Authors:** Isabel C. Caballero, Andrew J. Sakla, Jillian T. Detwiler, Marion Le Gall, Spencer T. Behmer, Charles D. Criscione

**Affiliations:** 1 Department of Biology, Texas A&M University, College Station, Texas, United States of America; 2 Department of Biological Sciences, University of Manitoba, Winnipeg, Canada; 3 Department of Entomology, Penn State University, State College, Pennsylvania, United States of America; 4 Department of Entomology, Texas A&M University, College Station, Texas, United States of America; Universidade de Aveiro, PORTUGAL

## Abstract

Negative effects of parasites on their hosts are well documented, but the proximate mechanisms by which parasites reduce their host’s fitness are poorly understood. For example, it has been suggested that parasites might be energetically demanding. However, a recent meta-analysis suggests that they have statistically insignificant effects on host resting metabolic rate (RMR). It is possible, though, that energetic costs associated with parasites are only manifested during and/or following periods of activity. Here, we measured CO_2_ production (a surrogate for metabolism) in Mediterranean geckos (*Hemidactylus turcicus*) infected with a lung parasite, the pentastome *Raillietiella indica*, under two physiological conditions: rested and recently active. In rested geckos, there was a negative, but non-significant association between the number of pentastomes (i.e., infection intensity) and CO_2_ production. In recently active geckos (chased for 3 minutes), we recorded CO_2_ production from its maximum value until it declined to a stationary phase. We analyzed this decline as a 3 phase function (initial decline, secondary decline, stationary). Geckos that were recently active showed, in the secondary phase, a significant decrease in CO_2_ production as pentastome intensity increased. Moreover, duration of the secondary phase showed a significant positive association with the number of pentastomes. These results suggest that the intensity of pentastome load exerts a weak effect on the metabolism of resting geckos, but a strong physiological effect on geckos that have recently been active; we speculate this occurs via mechanical constraints on breathing. Our results provide a potential mechanism by which pentastomes can reduce gecko fitness.

## Introduction

The ecological interspecific interaction of parasitism entails a benefit for the parasite while causing a negative fitness effect on their hosts. There are several examples where protozoan or metazoan parasites reduce the fitness of their hosts [1, 2]. However, the proximate mechanisms by which parasites may reduce host fitness are not well understood and can take on multiple forms. As consumers parasites exploit the host as a resource, but parasites can also extract energy from their hosts beyond what they directly consume [3]. For example, hosts may expend energy to resist or tolerate infection [4, 5] or use resources in order to recover or repair damage caused by parasites. These interactions will likely result in metabolic costs for the host [3], and recently this was investigated in a meta-analysis [6]. This analysis reported 22 studies that measured the impact of parasites on host resting metabolic rates (RMR). The meta-analysis included studies on various metazoan and protozoan parasites of vertebrate or invertebrate hosts. Of these, five studies showed a significant increase in RMR, four showed a significant decrease, and 13 were not significant. Thus, they concluded that there was no consistent generality with regards to the effect of parasites on host RMR. However, Robar, Murray [6] call attention to the fact that the metabolic impact of parasites on their hosts may not manifest unless the hosts are under stress (e.g., they are expending energy, perhaps as a function of defending territory, foraging for prey, or evading a predator).

To study the effects of parasitism on host metabolism, we used natural infections of an invasive pentastome parasite *Raillietiella indica* that infects the introduced Mediterranean gecko, *Hemidactylus turcicus*. Adult pentastomes are obligate parasites of the respiratory tract of their hosts (largely, reptilian) [7]. We used this system for three reasons. First, *R*. *indica* resides in the lungs, where they feed on blood. Moreover, lung function is likely impeded because *R*. *indica* is large (2.7–16.7 mm in length; Kelehear, Spratt [8]), and takes up physical space in the primary respiratory organ. Thus, a reasonable hypothesis is that *R*. *indica* would decrease the metabolic rate of its gecko host. Second, Pence and Selcer [9] provided evidence that *Raillietiella frenatus* [sic] (which is a junior synonym of *R*. *indica*, see Methods) negatively impacts the fitness of Mediterranean geckos in south Texas. As noted by Robar, Murray [6], there is a need to link the effects of parasites on host energy budgets to estimates of impacts on host fitness. Third, the Mediterranean gecko has a broad introduced distribution across much of the southern U.S. [10]. It is not uncommon for reptile and amphibian parasites to be generalists [11]. For example, the Mediterranean gecko in its introduced U. S. range has acquired several helminth parasites of native reptile or amphibian hosts [12, 13]. Because *R*. *indica* has been reported from several lizard and anuran species [14], there is the potential that the gecko could introduce this exotic parasite, with negative consequences, to native lizard fauna. Indeed, *R*. *frenatus* [sic] has been reported from introduced Hawaiian populations of green anoles, *Anolis carolinensis* [15]. We have recently documented, for the first time, *R*. *indica* infections in the native range of green anoles populations in Louisiana that are sympatric with infected Mediterranean gecko populations (unpublished data). Thus, it is important to determine if *R*. *indica* can cause negative physiological effects on lizard hosts in general. In this study, we tested the hypothesis that the effect of pentastomes on metabolic rate would be weak in rested geckos, but strong in geckos that were recently active. We did this using flow-through respirometry in two separate experiments. The first experiment measured CO_2_ production (a surrogate for metabolism) from geckos that were at rest, while the second measured CO_2_ production in geckos that were recently active (as a result of being chased for 3 minutes). Thus, by studying the impact of *R*. *indica* on the gecko’s metabolic rate under two different physiological conditions, we are able to address inconsistencies about predicting directionality of parasite effects on host metabolic rates (as reported in Robar, Murray [6]) and provide a test of a potential proximate mechanism that leads to the gecko’s reduced fitness.

## Materials and Methods

### Study system

We first note that *Raillietiella frenatus* was the pentastome identified in the Pence and Selcer [9] study. However, molecular and morphological data have indicated that *R*. *indica* and *R*. *frenatus* are the same species. Kelehear, Spratt [8] synonymized *R*. *indica* under *R*. *frenatus*. However, *R*. *indica* takes precedence in the literature [7]. Thus, we refer to the pentastome in our study and that of Pence and Selcer [9] as *R*. *indica*. Our own molecular and morphological data (unpublished) confirm the findings of Kelehear, Spratt [8]. *Raillietiella indica* has a natural distribution in Southeast Asia [7] and is thus considered to be an exotic species in the U.S. This pentastome has a complex life cycle where lizards and some amphibians are final hosts. Eggs are coughed up in mucus from the lower airways, or swallowed and passed in the feces. Cockroaches eat the eggs and serve as intermediate hosts. The life cycle is completed when the lizard host consumes the infected cockroach [16].

### Collecting geckos

We conducted two separate experiments to test the effect of infection on CO_2_ production. The first experiment, performed in 2012, tested the effect of parasites on the metabolic rate of rested geckos (*n* = 48). The second experiment, conducted in 2013, tested the effect of parasites on recently active geckos (*n* = 70). During September and October, geckos were sampled from two locations in southern Texas; the public grounds of the Marine Science Center in Port Aransas (27°50’11”N, 97°3’7”W) and the Lady of Assumption Church in Ingleside (27°52’3”N, 97°12’45”W). Geckos were captured by hand and fed a diet of grasshopper nymphs until experiments were performed. Geckos were dissected shortly after quantifying CO_2_ production and examined for the presence of metazoan parasites.

#### Ethics Statement

The Mediterranean gecko is an invasive species (non-endangered and not protected) in the United States and does not require collecting permits in the state of Texas. No permission was needed to sample from the Marine Science Center as this is public land. We obtained verbal permission from the administrative parish to sample from the Lady of Assumption Church. The research protocols [i.e., capture, handling, and sacrifice (decapitation followed by pithing) prior to dissection] in this study were approved by the Institutional Animal Care and Use Committee at Texas A&M University (AUP 2012–023).

### Measuring CO_2_

As a proxy for metabolism, we measured the rate of carbon dioxide production (VCO_2_). Following a 24h food deprivation (water was available via a dampened paper towel), we weighed geckos (± 0.1 mg), placed them in 50 mL (15 cm length x 2 cm depth) metabolic chambers and let them recover from handling for 1h before measurement commenced. All measurements were started between 1700 and 1900 h, because Mediterranean geckos become active in the early evening [17]. Most animals ceased movements within the first minute after insertion in the chamber and subsequently remained motionless. Chambers were held on a plastic rack over a heat pad (Zoo Med Labs Repti-Therm), and ambient temperature within the experimental chambers was maintained at 25°C using a temperature probe inside an empty chamber linked to the heat pad thermostat.

Dried, compressed air was flushed through the chambers at a rate of 3 mL.min^-1^ using a mass flow controller (MFS Sable System Las Vegas) with a control valve (Dylan Group). Upon leaving the chambers, the air was scrubbed of water vapor and carbon dioxide production of the geckos was determined by an infrared carbon dioxide analyzer (CA-10, 1 ppm resolution, Sable System Las Vegas). The analyzer provides fractional CO_2_ concentrations (parts per million), so we transformed the recording output using the following equation [18]:

$${\text{VCO}}_{2}{=\text{ FR}}_{\text{i }}\times \left({\text{F}}_{\text{e}}{\text{ CO}}_{2}{-\text{F}}_{\text{i}}{\text{CO}}_{2}\right),$$ (1)

where *F*_*e*_ *CO*_*2*_ = excurrent fractional concentration of CO_2_ leaving the chamber (here *F*_*i*_ *CO*_*2*_ = 0 because we used compressed air) and *FR*_*i*_ = incurrent flow rate (mL.min^-1^).

We used a multiplexer (RM-8 Flow Multiplexer, Sable System Las Vegas) so that five geckos could be processed simultaneously. VCO_2_ was recorded for bouts of 2 minutes for each gecko before switching to the next chamber. This was repeated five times so that the total recording lasted just under 1h. Thus, each gecko was recorded for a total of 10 minutes (2 min x 5 times). This allowed us to process all geckos within 3 days of capture. For our response variable in the statistical analyses on the metabolic rate of rested geckos, we averaged the VCO_2_ over the 10 min. Prior to averaging, we verified that VCO_2_ was stable over the time period using repeated measure analysis (data not shown).

In the second experiment, using geckos that were recently active, we followed the same protocol as above with the following exceptions: 1) The airflow rate in the system was increased to 31 mL.min^-1^ to be able to track fast changes in gecko metabolism after effort [18]. 2) To induce activity, individual geckos were placed in a 68 L plastic bin and then a single person, using one hand, chased the gecko for a period of 3 minutes. If geckos became exhausted before 3 minutes, they were gently finger tapped until the three-minute period ended. Geckos were then placed (individually) into a metabolism chamber and recording began immediately. VCO_2_ was recorded for a total of 1h. 3) Because we wanted to observe the changes in VCO_2_ production after effort, continuous recording was necessary. Thus, it was only possible to process one gecko at a time. Consequently, some geckos were held in captivity much longer (up to 20 days) than others. We controlled for this variable in our statistical analyses below.

### Statistical analysis

In all analyses, we performed general linear model tests of the relationship between production of CO_2_ and biologically relevant explanatory variables. Statistical analyses were done with R version 3.1.2 [19]. In the tests for rested geckos, our response variable was mean log_10_ (VCO_2_). Gecko body mass was used as a covariate. Because we used natural infections, we included several potential explanatory variables: gecko sex, population of capture (as a block), and infection intensities of several metazoan parasites. The parasites were an intestinal tapeworm (*Oochoristica javaensis*), an ectoparasitic mite (unidentified), encysted nematodes (unidentified), intestinal nematode A (unidentified pinworms), intestinal nematode B (unidentified), and pentastomes in the lung (*R*. *indica*).

In the recently active gecko experiment, there were a few changes to the explanatory variables. First, there were too few infections with the encysted nematodes and the intestinal nematode B (only two or three infections per species), so these were excluded from the model. Second, we added days-since-capture as a covariate to account for variation that may have been induced as a function of time in captivity. Also, the nature of the response variable in the stressed study was fundamentally different than that from the first experiment, using rested geckos. As noted above, VCO_2_ was averaged in the first experiment as it was stable over the measured period. In contrast, for recently active geckos, the rate of CO_2_ production after reaching a maximum decreased steeply during recovery and eventually reached an asymptote (Figure A in S1 File). Therefore, we used a Chi-square curve fitting method to describe this decline. To standardize among geckos, we analyzed the data by starting at the maximum VCO_2_. All geckos reached a stationary phase within an hour, thus we cut off the curves at time point 3453 sec (57.55 min) since the maximum. The following function was found to best describe the stressed gecko data.

$$\text{y }=\underset{\text{A}}{\underset{︸}{{\text{p}}_{1}{\;\times \text{ e}}^{-0.5\;{\left({\text{x}/\text{p}}_{2}\right)}^{2}}}}+\underset{\text{B}}{\underset{︸}{{\text{p}}_{3}{\;\times \text{e}}^{-\text{x}/{\text{p}}_{4}}}}+\underset{\text{C}}{\underset{︸}{{\text{p}}_{0}}}\;,$$ (2)

where *y* refers to VCO_2_ (mL.min^-1^) of stressed geckos and *x* is the time (s) since the maximum VCO_2_. The terms (*A*, *B*, and *C*) in eq (2) describe three phases of the decline in VCO_2_. Part *A* (initial phase) is represented by a Gaussian function and describes a rapid decline of respiration that occurs immediately after the maximum VCO_2_. Part *B* (secondary phase) corresponds to an exponential function and describes the decline of respiration towards a stationary state. Part *C* (stationary phase) is described by a constant parameter *p*_*0*_ (Figure A in S1 File). Parameters *p*_*0*_, *p*_*1*_ and *p*_*3*_ represent the VCO_2_ produced during the stationary, initial and secondary phases, respectively, while the parameters *p*_*2*_ and *p*_*4*_ correspond to the time a stressed gecko spends in the initial and secondary phases, respectively. These five parameters were used as response variables in the analyses for the stressed geckos. Because this represents multiple testing, we Bonferroni corrected our P-value cutoff (significance deemed at p ≤ 0.01) [20].

We started analyses by fitting full models to the data including all the above-mentioned explanatory variables. We note that the samples sizes were too small to test all potential interactions because the models would be over parameterized. We did explore two-way interactions involving pentastomes only; however, none of these interactions were significant in either the first or second experiment. We also used an automated backward stepwise algorithm to find minimal models based on Akaike Information Criterion (AIC, Akaike [21]) values. This process entailed removing the non-significant explanatory variables starting with the least significant term. Relationships between variables are shown using partial regression plots that display information about a single regression coefficient (from the explanatory variable of interest) after removing the effects of the remaining explanatory variables on both the response variable and the explanatory variable of interest [22]. We inspected full and minimal models for outliers and corrected departures of normality by log_10_-transforming variables. Normality was tested with the Lilliefors test [23] as implemented in R. All model residuals were visually checked for homoscedasticity.

## Results

**Fig 1:**
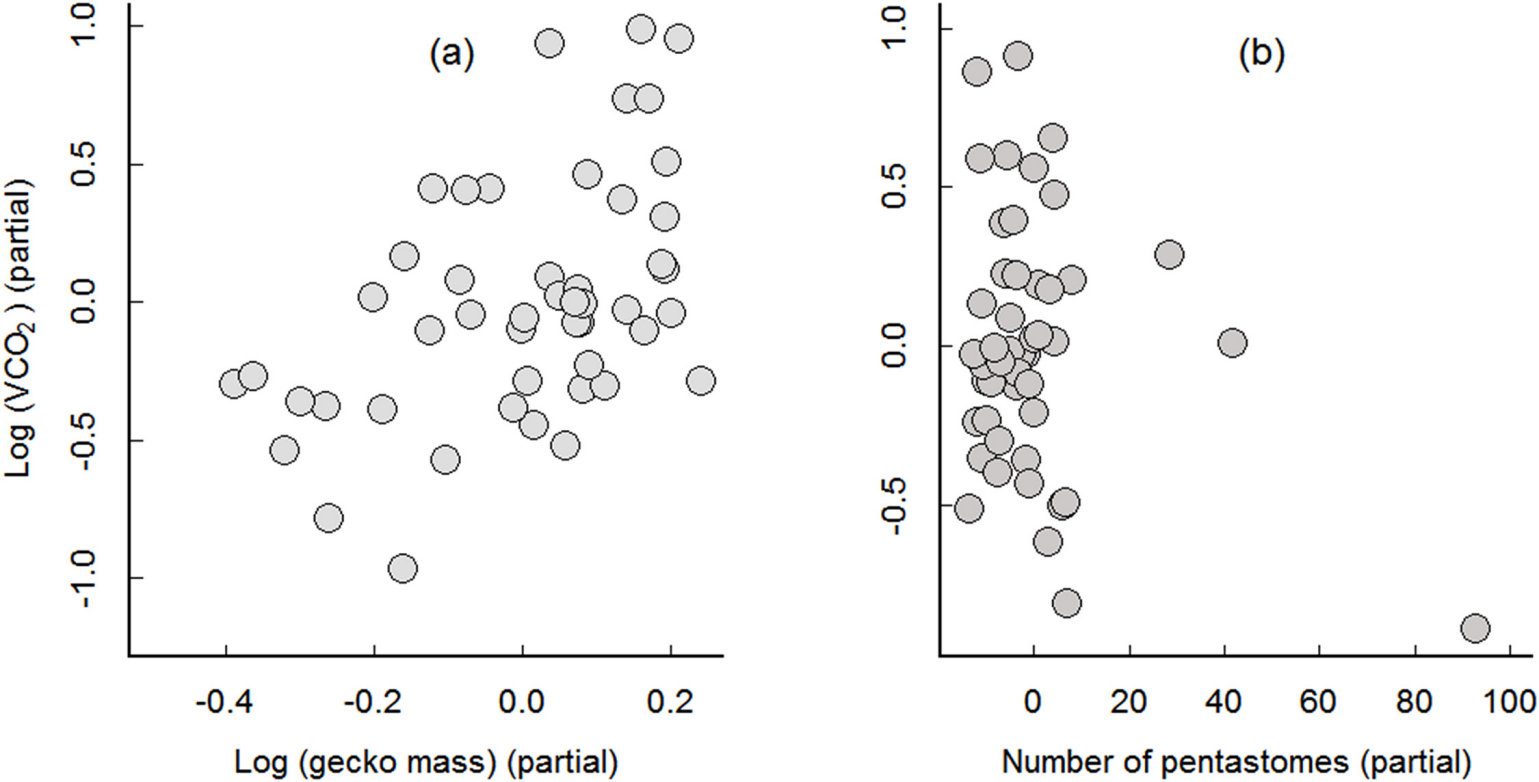
Relationship between mass and number of pentastomes versus log (VCO_2_) production for rested geckos. Partial regression plots (*y* axis: residuals of regressing the response variable on the explanatory variables, but omitting the explanatory variable of interest, and *x* axis: residuals of each explanatory variable of interest regressed on all remaining explanatory variables) of (a) the relationship of log (gecko mass) to log (VCO_2_), given the effect of number of pentastomes, (*n* = 48); and (b) the relationship of number of pentastomes to log (VCO_2_) given the effect of log (gecko mass). Minimal regression model for the rested gecko experiment: log (VCO_2_) = (1.023 × log (gecko mass))–(0.005 × number of pentastomes)– 2.465; model adjusted *R* ^*2*^ = 0.2093.

The results of the full models and minimal models are shown in Tables 1 and 2, respectively. In the experiment using rested geckos, the full model showed only gecko mass as having a significant positive relationship with VCO_2_. There was a negative relationship between the intensity of pentastomes and VCO_2_, but it was non-significant (P = 0.087) (Table 1 and Table B in S2 File). The minimal model leads to the same conclusion where the effect of pentastomes remains non-significant (P = 0.072) (Table 2 and Table C in S2 File). The residual relationships with gecko body mass and number of pentastomes to VCO_2_ is shown in Fig 1.

**Table 1:** Effects of explanatory variables on VCO_2_ from rested geckos (*n* = 48) and recently active geckos (*n* = 70) analyzed by linear models. The same explanatory variables were used to assess time to recovery in recently active geckos. The P*-*values associated with significant predictors are in bold. F-values for these tests are reported in Table B in S2 File.

Study	Rested	Recently active				
Phase		Stationary	Initial		Secondary	
Response variable (parameters)	VCO_2_ ^a^	VCO_2_ (*p*_*0*_)	VCO_2_ (*p*_*1*_)	Time (*p*_*2*_)	VCO_2_ (*p*_*3*_)	Time^a^ (*p*_*4*_)
Predictor variable						
Sex	0.8581	NA	NA	NA	NA	NA
Population	0.9098	**0.0067**	0.0846	0.2376	0.5614	0.2926
Days after capture	NA	0.6781	0.3736	0.3461	0.5284	0.2444
Gecko mass^b^	**0.0072**	**< 0.0001**	0.2275	0.2391	**< 0.0001**	0.9183
Ectoparasitic mites (unidentified)	0.6520	0.5115	0.2041	0.7677	0.0700	0.9305
Pentastomes (*R*. *indica*)	0.0874	0.1475	0.1723	0.9536	**0.0044**	**0.0009**
Intestinal tapeworms (*O*. *javaensis*)	0.1014	0.6920	0.4672	0.4029	0.8238	0.9518
Intestinal nematodes A (unidentified pinworms)	0.4313	0.2683	0.9657	0.9145	0.4053	0.2596
Encysted nematodes (unidentified)	0.135	NA	NA	NA	NA	NA
Intestinal nematode B (unidentified)	0.376	NA	NA	NA	NA	NA

^a^Variable was log-transformed

^b^Mass was log-transformed only for the resting study

NA = variable not included in analyses, see explanation in text

**Table 2:** Effect of explanatory variables on VCO_2_ found in minimal models obtained by an automated stepwise backward procedure based on AIC values for rested geckos (*n* = 48) and recently active geckos (*n* = 70). The same explanatory variables were used to assess time to recovery in recently active geckos. The excluded explanatory variables by the automated procedure are denoted with a dash symbol. The P-values associated with significant predictors are in bold. F-values for these tests are reported in Table C in S2 File.

Study	Rested	Recently active				
Phase		Stationary	Initial		Secondary	
Response variable	VCO_2_ ^a^	VCO_2_	VCO_2_	Time	VCO_2_	Time^a^
(parameters)		(*p*_*0*_)	(*p*_*1*_)	(*p*_*2*_)	(*p*_*3*_)	(*p*_*4*_)
Predictor variable						
Sex	–	NA	NA	NA	NA	NA
Population	–	**0.0012**	**0.0067**	–	–	–
Days after capture	NA	–	–	–	–	–
Gecko mass^b^	**0.0006**	**<0.0001**	0.0819	0.0545	**< 0.0001**	–
Ectoparasitic mites (unidentified)	–	–	–	–	0.0508	–
Pentastomes (*R*. *indica*)	0.0716	–	–	–	**0.0016**	**0.0008**
Intestinal tapeworms (*O*. *javaensis*)	0.0991	–	–	–	–	–
Intestinal nematodes A (unidentified pinworms)	–	–	–	–	–	–
Encysted nematodes (unidentified)	0.1087	NA	NA	NA	NA	NA
Intestinal nematode B (unidentified)	–	NA	NA	NA	NA	NA

^a^ Variable was log-transformed

^b^ Gecko mass was log-transformed only for the resting study

NA = variable not included in analyses, see explanation in text

In the experiment using recently active geckos, 10 geckos were, for uncontrollable reasons, not sexed. Thus, we analyzed the data with and without host sex in the model. Qualitatively, we found similar results as host sex does not test significant (as was also the case for rested geckos). Thus, we focused on the analyses that excluded host sex (Tables 1 and 2), but have provided the results of the full model when host sex was included (Table A and Table D in S2 File).

**Fig 2:**
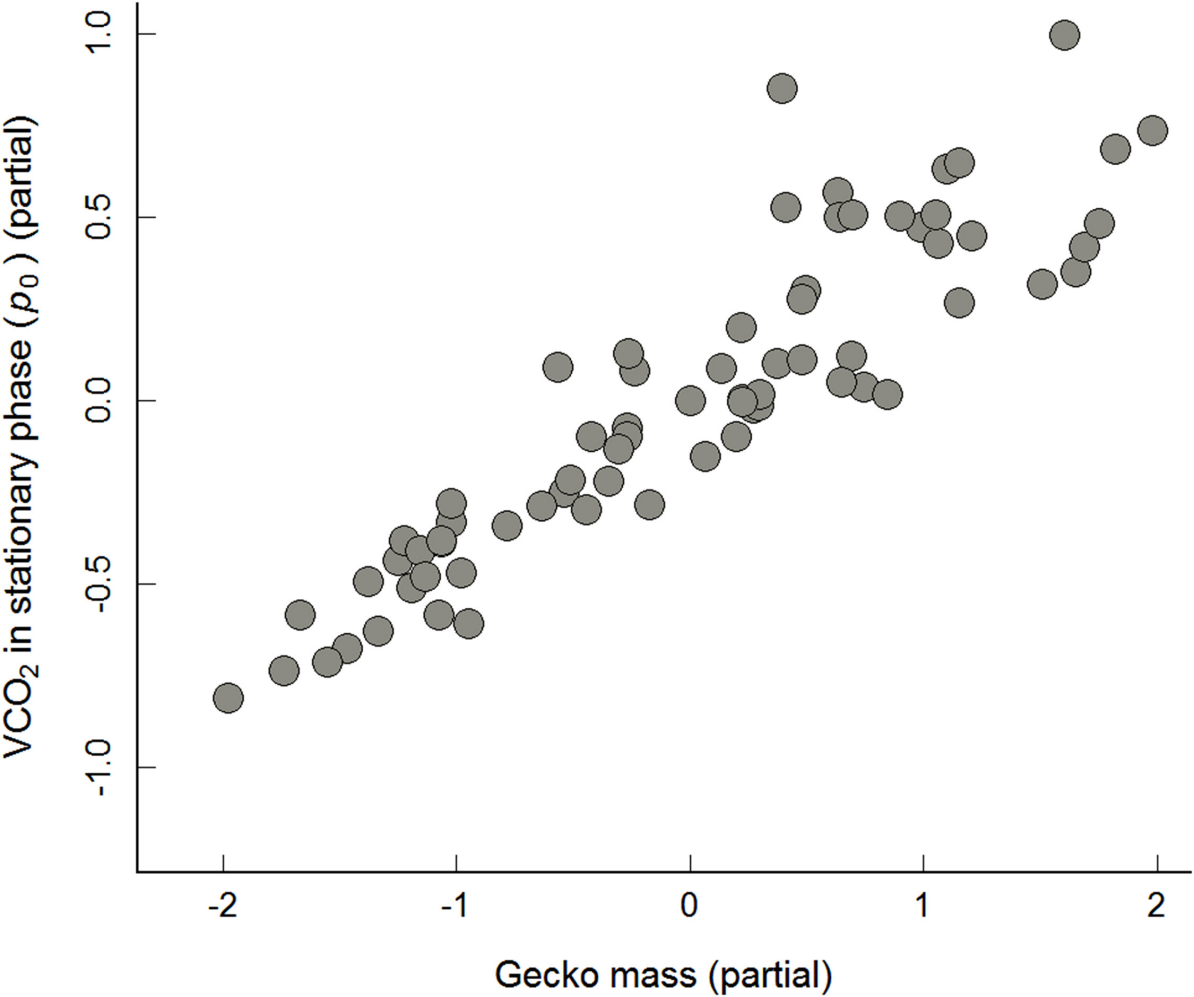
Relationship between mass versus VCO_2_ production in stationary phase (*p*_*0*_) for recently active geckos. Partial regression plot (*y* axis: residuals of regressing the response variable on the explanatory variables, but omitting the explanatory variable of interest, and *x* axis: residuals of each explanatory variable of interest regressed on all remaining explanatory variables) of the relationship of gecko mass to VCO_2_ produced in stationary phase (*p*_*0*_) for recently active geckos (*n* = 70) given the effect of population. Minimal regression model for recently active geckos: VCO_2_ (*p*_*0*_) = (0.390 × gecko mass)– 0.101; model adjusted *R* ^*2*^ = 0.8239.

**Fig 3:**
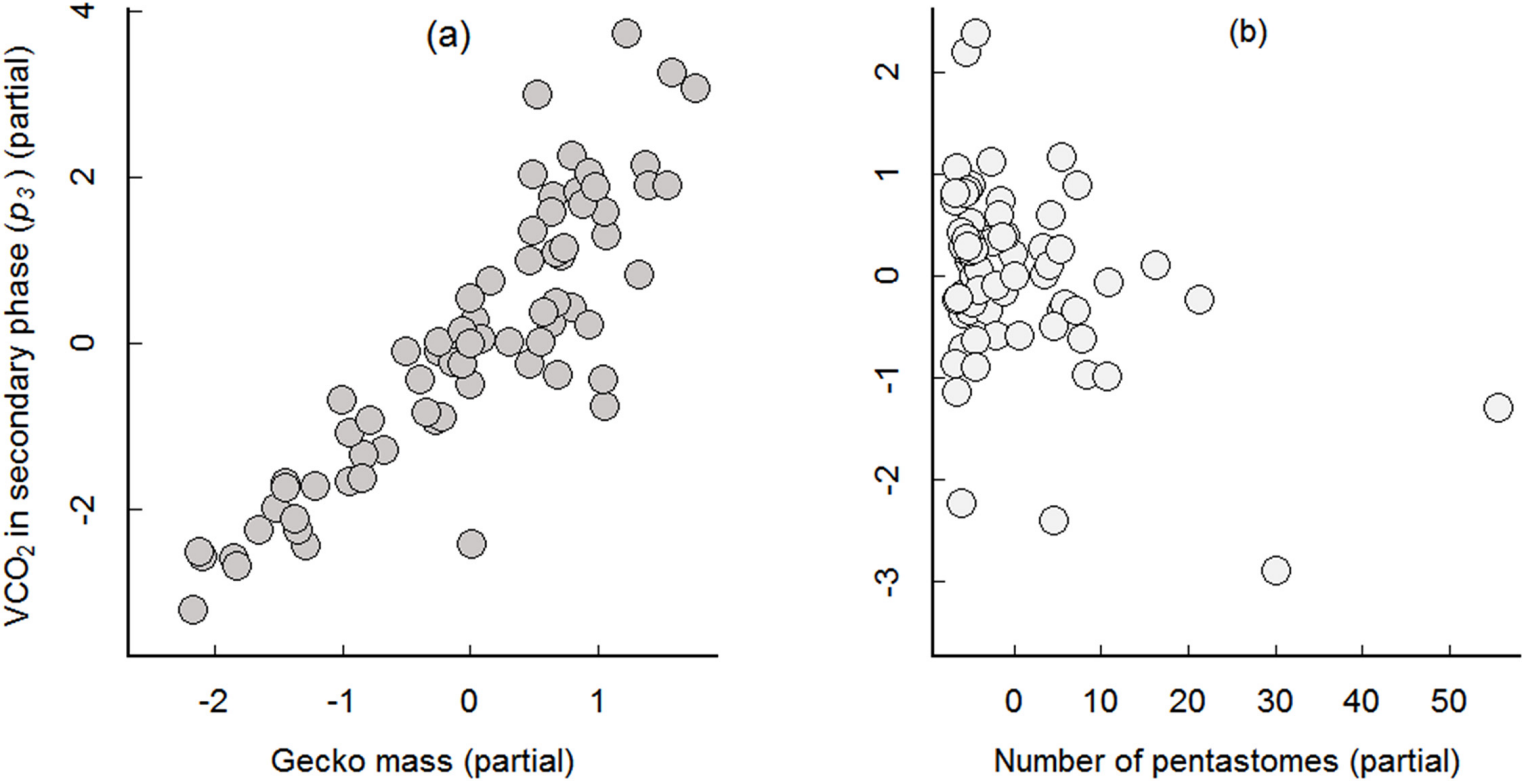
Relationship between mass and number of pentastomes versus VCO_2_ production in secondary phase (*p*_*3*_) for recently active geckos. Partial regression plots (*y* axis: residuals of regressing the response variable on the explanatory variables, but omitting the explanatory variable of interest, and *x* axis: residuals of each explanatory variable of interest regressed on all remaining explanatory variables) of (a) the relationship of gecko mass to VCO_2_ produced in secondary phase (*p*_*3*_) for recently active geckos (*n* = 70) given the effect of number of pentastomes and mites, and (b) the relationship of number of pentastomes to CO_2_ produced given the effect of gecko mass (g) and mites. Minimal regression model for recently active geckos: VCO_2_ (*p*_*3*_) = (1.411 × gecko mass)–(0.033 × number of pentastomes)–(0.063 × number of mites) + 0.141; model adjusted *R* ^*2*^ = 0.7649.

**Fig 4:**
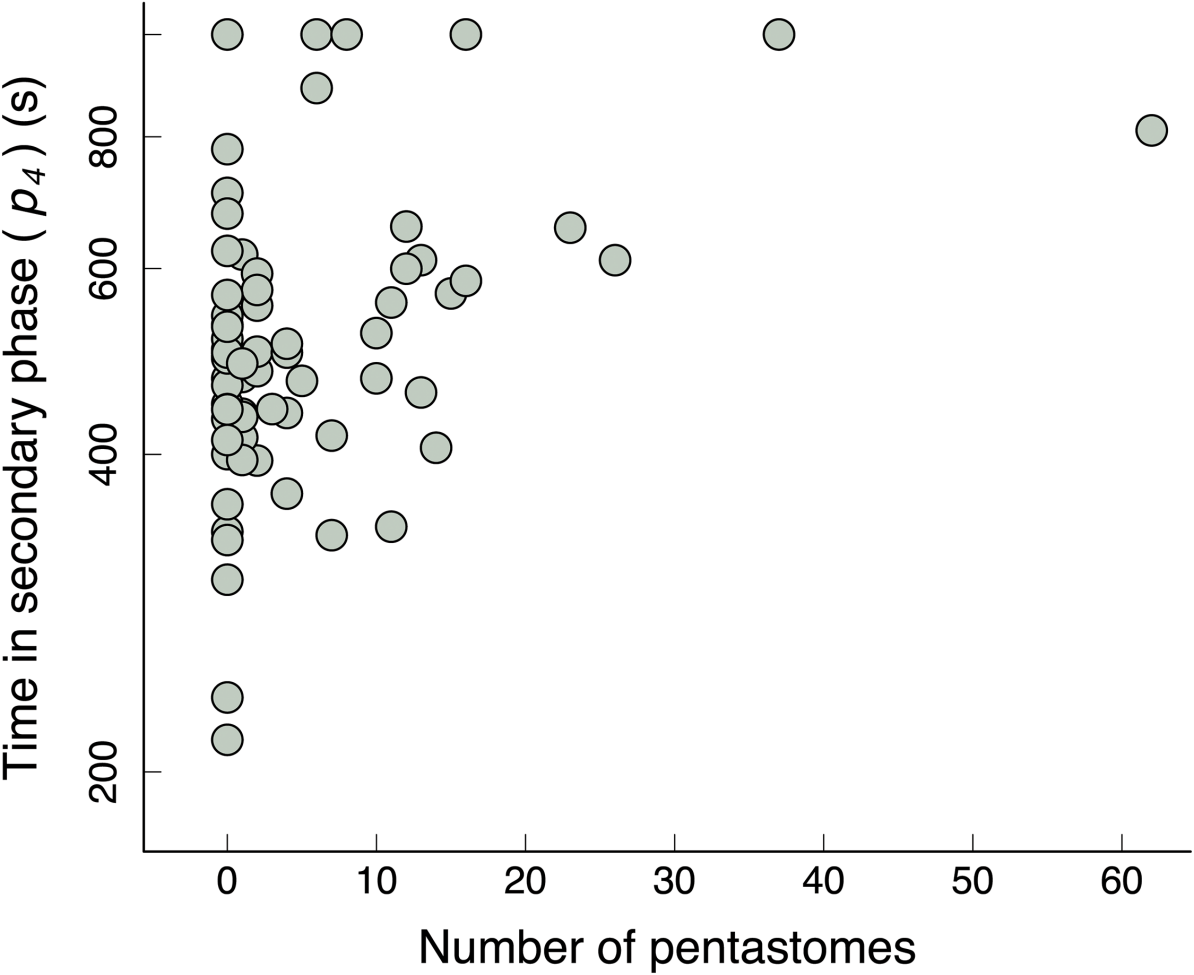
Relationship between time in secondary phase (*p*_*4*_) versus number of pentastomes for recently active geckos. Minimal regression model for recently active geckos (*n* = 70): log (time in stationary phase) (*p*_*4*_) = (0.013 × number of pentastomes) + 6.164; model adjusted *R* ^*2*^ = 0.1424. Note log scale used on y-axis.

In the full and minimal models, gecko body mass had a significant positive relationship with VCO_2_ in the stationary phase (*p*_*0*_) and in the secondary phase (*p*_*3*_) (Tables 1 and 2), indicating that heavier geckos produced more VCO_2_ than lighter geckos (partial plots showing positive relationships with *p*_*0*_ and *p*_*3*_ are given in Figs 2 and 3a). In contrast to the tests using rested geckos, we observed a significant effect of pentastome intensity on the production of VCO_2_ in recently active geckos. This effect occurred during the secondary phase (*p*_*3*_) (Tables 1 and 2 for the full and minimal models, respectively). Moreover, time spent in the secondary phase (*p*_*4*_) also tested significant with the intensity of pentastome infection in the full and minimal models (Tables 1 and 2). Partial plots showed that there was a negative relationship between pentastome intensity and production of VCO_2_ (Fig 3b), but a positive relationship between pentastome intensity and time in the secondary phase (*p*_*4*_) (Fig 4).

We recognized that a few heavily infected geckos might be driving our results. Therefore, we checked the Cook’s distance measures for the heavily infected residual data points in both data sets (rested and recently active geckos). This analysis showed no significant influence of these data points. In addition, previous studies have shown that co-infections by multiple parasite species could impact host physiology (e.g., [24]). Our results do not appear to be influenced by co-infections as we did not detect any significant pairwise interactions involving pentastomes and the other parasites. Moreover, post-hoc analyses where parasite richness (total number of parasite species in an individual host) was used as the explanatory variable in place of individual parasite infection intensities did not shown significance for parasite richness in any of the models discussed above (data not shown). Thus, it appears in our study system that co-infections are not a major driver affecting host metabolism.

## Discussion

Our experiments, using field-collected geckos, provide evidence that pentastome lung infections have the effect of significantly reducing gecko CO_2_ production, and thus by extension metabolic rate. However, this effect was only observed in geckos that had recently been active.

In the rested geckos, CO_2_ production decreased as pentastome intensity increased (Fig 1b), but this relationship was non-significant (Tables 1 and 2). A few potential explanations (not necessarily mutually exclusive) might explain the lack of significance. First, pentastomes are known to secrete surfactants [25]. Lower tetrapods secrete pulmonary surfactants to prevent lung collapse and in mammals, pulmonary surfactants regulate immune activity [25]. The pentastome surfactants may enable immune evasion and reduce inflammation of the lung tissue. Thus, resting geckos may not be expending energy on either immune defense (as the parasite is “hidden”) or tissue repair. Second, it may be that the basal metabolic needs of resting geckos are low and the amount of blood taken from the host by the parasite, even at higher intensity, is small enough that it does not impact gecko metabolic rate in rested geckos. Third, metazoan parasites in general show aggregated distributions among their hosts [26, 27]. Indeed this is what we observed (i.e., most hosts are uninfected or have low intensity infections and very few hosts have high intensity infections) in both sampling collections for the two studies (Figure B in S1 File). Thus, with few high intensity infections, there could be a lack of statistical power.

In the experiments using recently active geckos, we examined five parameters associated with the decline in VCO_2_ from its maximum. In the initial phase, we did not detect significance for any of the explanatory variables, including gecko body mass. This phase may be too short or lack enough variation to detect any statistical differences. In contrast, in both the secondary phase and stationary phase, gecko body mass was positively associated with VCO_2_ (*p*_*3*_ in Fig 3a and *p*_*0*_ in Fig 2, respectively). The positive association with body mass is as expected (see Lighton [18]), and is consistent with the one observed in the rested gecko experiment. However, analysis of the secondary phase showed that higher pentastome intensities in the lungs were significantly correlated with lower VCO_2_; this occurred even after Bonferroni correction (*p*_*3*_, Tables 1 and 2). The lower VCO_2_ in higher intensity infections may be related to physical obstructions caused by the pentastomes. The lungs of lizards are simple sacs and the respiratory area is small [28]. For example, pulmonary capacity (tidal volume) may be reduced due to engorgement of the lungs, which has been observed in heavily infected geckos [9]. In addition, running geckos are mechanically constrained and the ventilation of their lungs compromised because hypaxial muscles are used intermittently for both locomotion and breathing [29–32]. Having the additional burden of pentastomes within the lungs while running may further tax muscle function of the lungs and hence decrease downstream metabolism. It may also be that while lost blood during resting does not impact host metabolism, the prior loss of blood may influence metabolism after a stress event. Interestingly, there was no effect of pentastome infections on metabolism in the stationary phase. This result is consistent with the VCO_2_ levels observed in the rested geckos and suggests pentastomes do not cause a major metabolic cost to the host while it is at rest.

If the pentastomes are affecting VCO_2_ levels, there will likely be effects of the pentastome infection on gecko energy budgets. For example, if infected geckos that have recently been active cannot respire as efficiently as uninfected geckos that have recently been active, then ATP production will be reduced, and an infected gecko may take longer to return to a stable rate of respiration. Indeed, in addition to the reduced VCO_2_ output in the secondary phase, higher pentastome intensities were also positively correlated with time that geckos spent in the secondary phase.

An alternative way of assessing the impact of parasites on the metabolic rate of recently active hosts is the use of maximum VCO_2_, which is analogous to the maximal metabolic rate reported in other respirometry studies testing the effects of parasites [33–37]. Maximal metabolic rate is associated with high-energy expenditure activities such as foraging, escaping from predators, running stamina, and defense of territories [38]. To provide a comparative basis to other studies, we conducted a *post hoc* analysis of the recently active gecko data using the maximum VCO_2_ as a response variable. We note, however, that in the curve fitting method at time 0 (*x* = 0), the maximum VCO_2_ is the sum of *p*_*0*_, *p*_*1*_ and *p*_*3*_ (*see* eq (2)), and is therefore not an independent analysis of what we reported above. Using the maximum VCO_2_, gecko body mass had a significant positive association (P < 0.001) and the number of pentastomes had a significant negative association (P = 0.006) with maximum VCO_2_. This result was similar to that obtained from the secondary phase (*p*_*3*_) parameter (with the difference in that the curve-fitting method provides additional information on time spent in recovery). Including our study with prior studies that stressed/worked their hosts, three showed a significant decrease in host metabolism for infected hosts [34, 37, this study] and three showed no effect [33, 35, 36]. Currently there are too few studies that use maximal metabolic rate to make a global conclusion on the effects of parasites on recently active/stressed hosts.

The meta-analysis of Robar, Murray [6] only considered infection status (infected or not) of the hosts. They acknowledged that an analysis of parasite intensities on host RMR would be valuable, but that they were unable to extract the necessary data from the literature. Our study provides evidence that an increase in pentastome intensity, but not intensities of the other observed metazoan parasites, decreases the metabolic rate of recently active geckos. In addition, Robar, Murray [6] emphasized that relating energy perturbations due to parasitism with estimates of host fitness will increase our understanding of parasite effects on host energy budgets. The association of pentastome infections and fitness of the Mediterranean geckos was tested by Pence and Selcer [9]. They found that heavily infected geckos with pentastomes in southern Texas had significant reductions in fatbodies during the reproductive period, and female geckos had lower liver mass during the non-reproductive period. Moreover, heavily infected females had a lower percentage of oviductal eggs compared to those of uninfected females [9]. The results of our study provide a potential mechanism by which gecko fitness is reduced by pentastomes. Decreased gaseous exchanges as a result of being infected and active (e.g., fleeing predators such as domestic cats) will reduce cellular respiration and ATP production. Then, these decreased gaseous exchanges may be energetically costly for basic physiological needs. Moreover, a prolonged recovery time after being stressed could decrease foraging time, and hence fatbody stores, for infected geckos.

In summary, we found a negative, but non-significant association between number of pentastomes and production of VCO_2_ when geckos were in a resting state. However, we found a significant negative association between pentastome intensity and VCO_2_ production in geckos that had recently been active. Thus, our study highlights the need for considering physiological states other than resting when evaluating how different biological factors (parasites, toxins, etc.) can affect metabolism. By curve fitting the decline in VCO_2_, we were able to extract additional information concerning recovery time. In particular, there was a significant positive association between time spent recovering towards a stationary state and pentastome intensity. The decreased metabolism and prolonged recovery after a period of sustained activity provides a potential mechanism by which gecko fitness is reduced by pentastomes [9]. It will be interesting, and of conservation importance to ascertain if similar metabolic costs are experienced by green anoles in native populations that we recently found to also be infected with the pentastome *R*. *indica* (unpublished).

## Supporting Information

S1 File Contains Figure A in S1 File. Parameterization of the decline of VCO_2_ production. Figure B in S1 File. Observed pentastome frequency distributions among rested geckos and recently active geckos.
(DOCX)

S2 File Contains Table A in S2 File. Effects of explanatory variables (including sex) on VCO_2_ in recently active geckos. Table B in S2 File. F-values corresponding with models detailed in Table 1. Table C in S2 File. Summary of explanatory variables and F-values on VCO_2_ for rested geckos and recently active geckos from minimum models detailed in Table 2. Table D in S2 File. Summary of explanatory variables and F-values on VCO_2_ for recently active geckos detailed in Table A in S2 File.
(DOCX)
