# Supplementary material for: Physiological Status Drives Metabolic Rate in Mediterranean Geckos Infected with Pentastomes
Source: PLoS One. 2015 Dec 14;10(12):e0144477. doi: 10.1371/journal.pone.0144477 (PMC4681768; doi:10.1371/journal.pone.0144477)

Supplementary Figures (S1 File)

**Physiological Status Drives Metabolic Rate in Mediterranean Geckos Infected with Pentastomes**

Isabel. C. Caballero^*^, Andrew J. Sakla, Jillian T. Detwiler, Marion Le Gall, Spencer T. Behmer, Charles D. Criscione

*Correspondence: [icabal@tamu.edu](mailto:icabal@tamu.edu) (ICC)

**
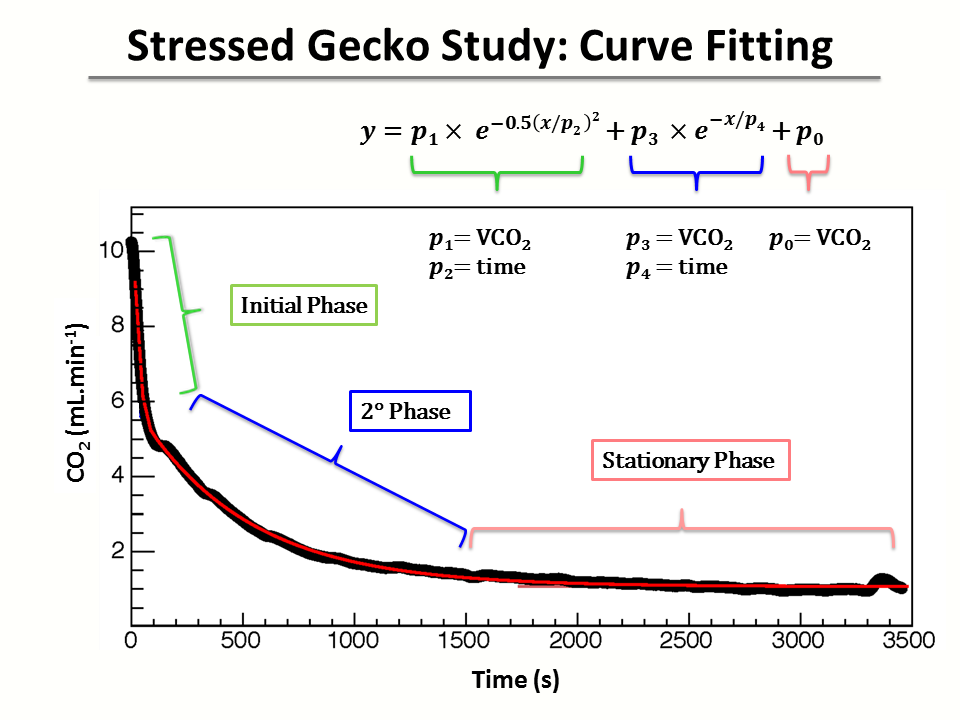
Figure A in S1 File**. **Parametrization of the decline of VCO_2_ production**. This parametrization was carried out in recently active Mediterranean geckos, by Chi-square curve fitting from a random gecko host (the black curve shows the respirometry data points). A curve was fitted to the data (shown as the red line) using a function with three terms that described three phases of the decline in VCO_2_ since the maximum. The initial phase starts after the maximum VCO_2_ and is described by a Gaussian function with the parameters *p_1_* (the amount of VCO_2_ in the initial phase) and *p_2_* (time spent in the initial phase). In the secondary phase VCO_2_ continues to decline, but is described by an exponential function with the parameters *p_3_* (the amount of VCO_2_ in the secondary phase) and *p_4_* (time spent in the initial phase). The stationary phase is described by the constant parameter *p_0_*. Brackets denote approximate breakpoints for the phases.

**Figure B in S1 File. Observed pentastome frequency distributions among rested geckos and recently active geckos**.

Pentastome frequency distributions among Mediterranean geckos included in (a) the rested gecko experiment (2012), and (b) the recently active gecko experiment (2013).


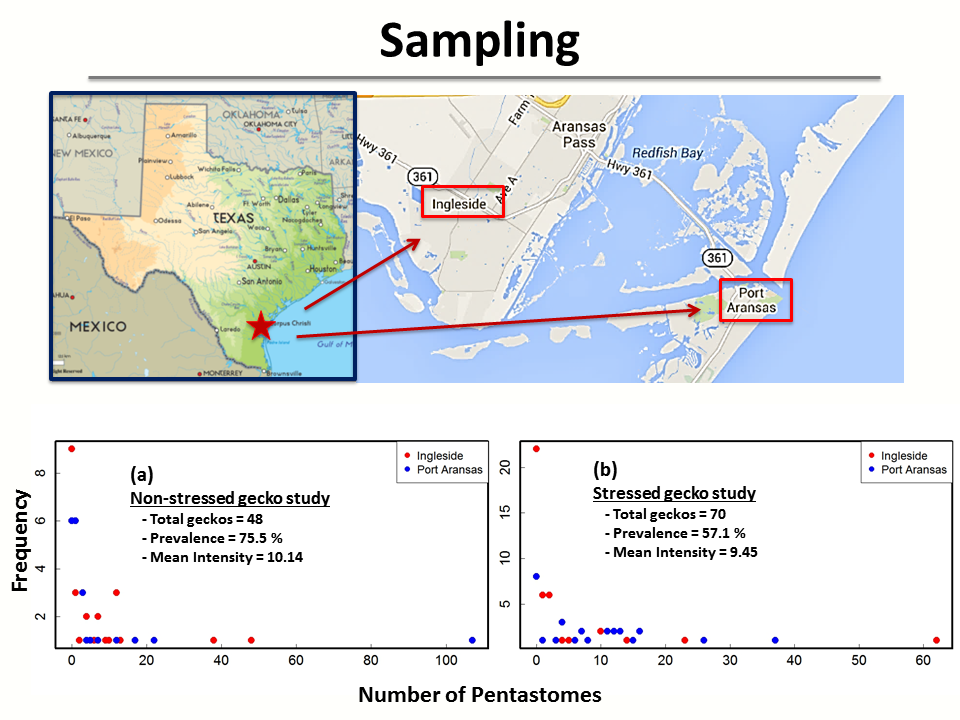

Supplement: S1 File — Parameterization of the decline of VCO2 production. Figure B in S1 File. Observed pentastome frequency distributions among rested geckos and recently active geckos. (DOCX) [file pone.0144477.s001.docx]
