# Supplementary material for: Physiological Status Drives Metabolic Rate in Mediterranean Geckos Infected with Pentastomes
Source: PLoS One. 2015 Dec 14;10(12):e0144477. doi: 10.1371/journal.pone.0144477 (PMC4681768; doi:10.1371/journal.pone.0144477)
Supplement: S2 File — Effects of explanatory variables (including sex) on VCO2 in recently active geckos. Table B in S2 File. F-values corresponding with models detailed in Table 1. Table C in S2 File. Summary of explanatory variables and F-values on VCO2 for rested geckos and recently active geckos from minimum models detailed in Table 2. Table D in S2 File. Summary of explanatory variables and F-values on VCO2 for recently active geckos detailed in Table A in S2 File. (DOCX) [file pone.0144477.s002.docx]

Supplementary Tables (S2 File)

**Physiological Status Drives Metabolic Rate in Mediterranean Geckos Infected with Pentastomes**

Isabel C. Caballero^*^, Andrew J. Sakla, Jillian T. Detwiler, Marion Le Gall, Spencer T. Behmer, Charles D. Criscione

*Correspondence: [icabal@tamu.edu](mailto:icabal@tamu.edu) (ICC)

**Table A in S2 File**. Effects of explanatory variables (including sex) on VCO_2_ in recently active geckos (*n* = 60). The P-values associated with significant predictors are in bold. F-values for these tests are reported in Table D in S2 File..

| Phase | Stationary |  | Initial | |  | Secondary | |
| --- | --- | --- | --- | --- | --- | --- | --- |
| Response variables | VCO_2_ |  | VCO_2_ | Time |  | VCO_2_ | Time^a^ |
| (parameters) | (*p_0_*) |  | (*p_1_*) | (*p_2_*) |  | (*p_3_*) | (*p_4_*) |
| Predictor variables |  |  |  |  |  |  |  |
| Sex | 0.5327 |  | 0.1868 | 0.1767 |  | 0.7875 | 0.7299 |
| Population | 0.0162 |  | 0.0957 | 0.1802 |  | 0.5556 | 0.5889 |
| Days after capture | 0.5960 |  | 0.2210 | 0.8711 |  | 0.5706 | 0.3882 |
| Gecko mass | **<0.0001** |  | 0.4357 | 0.2796 |  | **<0.0001** | 0.4259 |
| Ectoparasitic mites (unidentified) | 0.5226 |  | 0.3297 | 0.6256 |  | 0.1523 | 0.7575 |
| Pentastomes (*R. indica*) | 0.1573 |  | 0.2539 | 0.9168 |  | 0.0115 | **0.0053** |
| Intestinal tapeworms (*O. javaensis*) | 0.9233 |  | 0.5924 | 0.5274 |  | 0.8870 | 0.9195 |
| Intestinal nematodes A (unidentified pinworms) | 0.3243 |  | 0.9270 | 0.9180 |  | 0.5091 | 0.2744 |
|  |  |  |  |  |  |  |  |

^a^Variable was log-transformed

| Study | Rested |  | Recently Active | | | | | | |
| --- | --- | --- | --- | --- | --- | --- | --- | --- | --- |
| Phase |  |  | Stationary |  | Initial | |  | Secondary | |
| Response variable | VCO_2_^a^ |  | VCO_2_ |  | VCO_2_ | Time |  | VCO_2_ | Time^a^ |
| (parameters) |  |  | (*p_0_*) |  | (*p_1_*) | (*p_2_*) |  | (*p_3_*) | (*p_4_*) |
| Explanatory variables |  |  |  |  |  |  |  |  |  |
| Sex | 0.0324 |  | NA |  | NA | NA |  | NA | NA |
| Population | 0.0130 |  | 7.8657 |  | 3.0718 | 1.4223 |  | 0.3410 | 0.5821 |
| Days after capture | NA |  | 0.1739 |  | 0.8033 | 0.9014 |  | 0.4020 | 0.9564 |
| Gecko mass^b^ | 8.0774 |  | 227.1483 |  | 1.4855 | 1.4130 |  | 126.7495 | 0.1155 |
| Ectoparasitic mites (unidentified) | 0.2066 |  | 0.4361 |  | 1.6476 | 0.0880 |  | 3.3995 | 0.4366 |
| Pentastomes (*R. indica*) | 3.0793 |  | 2.1513 |  | 1.9070 | 0.0034 |  | 8.7407 | 13.8512 |
| Intestinal tapeworms (*O. javaensis*) | 2.8183 |  | 0.1584 |  | 0.5352 | 0.7092 |  | 0.0500 | 0.0895 |
| Intestinal nematodes A (unidentified pinworms) | 0.6327 |  | 1.2477 |  | 0.0019 | 0.0116 |  | 0.7021 | 1.1757 |
| Encysted nematodes (unidentified) | 2.3285 |  | NA |  | NA | NA |  | NA | NA |
| Intestinal nematode B (unidentified) | 0.8039 |  | NA |  | NA | NA |  | NA | NA |
| df (Error) | 38 |  | 62 |  | 62 | 62 |  | 62 | 62 |

**Table B in S2 File**. Summary of explanatory variables and their F-values on VCO_2_ for rested geckos and VCO_2_ and time for recently active geckos analyzed by linear models (*see* Table 1). All explanatory variables had 1 degree of freedom (df). The df for each model error is given in the final row.

^a^Variable was log-transformed

^b^Mass was log-transformed only for the resting study

NA= variable not included in analyses, see explanation in text

| Study | Rested |  | Recently active | | | | | | |
| --- | --- | --- | --- | --- | --- | --- | --- | --- | --- |
| Phase |  |  | Stationary |  | Initial | |  | Secondary | |
| Response variable | VCO_2_^a^ |  | VCO_2_ |  | VCO_2_ | Time |  | VCO_2_ | Time^a^ |
| (parameters) |  |  | (*p_0_*) |  | (*p_1_*) | (*p_2_*) |  | (*p_3_*) | (*p_4_*) |
| Explanatory variable |  |  |  |  |  |  |  |  |  |
| Sex | – |  | NA |  | NA | NA |  | NA | NA |
| Population | – |  | 11.422 |  | 7.8331 | – |  | – | – |
| Days after capture | NA |  | – |  | – | – |  | – | – |
| Gecko mass^b^ | 13.5759 |  | 324.5020 |  | 3.1207 | 3.8280 |  | 222.4934 | – |
| Ectoparasitic mites (unidentified) | – |  | – |  | – | – |  | 3.9574 | – |
| Pentastomes (*R. indica*) | 3.4111 |  | – |  | – | – |  | 10.8816 | 12.4540 |
| Intestinal tapeworms (*O. javaensis*) | 2.8411 |  | – |  | – | – |  | – | – |
| Intestinal nematodes A (unidentified pinworms) | – |  | – |  | – | – |  | – | – |
| Encysted nematodes (unidentified) | 2.6830 |  | NA |  | NA | NA |  | NA | NA |
| Intestinal nematode B (unidentified) | – |  | NA |  | NA | NA |  | NA | NA |
| df (Error) | 43 |  | 67 |  | 67 | 68 |  | 66 | 68 |

**Table C in S2 File**. Summary of explanatory variables and their F-values on VCO_2_ for rested geckos and VCO_2_ and time for recently active geckos from minimum models (*see* Table 2). These models were obtained by an automated stepwise backward procedure based on AIC values. All explanatory variables had 1 degree of freedom (df). The df for each model error is given in the final row.

^a^Variable was log-transformed

^b^Mass was log-transformed only for the resting study

NA= variable not included in analyses, see explanation in text

| Phase | Stationary |  | Initial | |  | Secondary | |
| --- | --- | --- | --- | --- | --- | --- | --- |
| Response variables | VCO_2_ |  | VCO_2_ | Time |  | VCO_2_ | Time^a^ |
| (parameters) | (*p_0_*) |  | (*p_1_*) | (*p_2_*) |  | (*p_3_*) | (*p_4_*) |
| Explanatory variables |  |  |  |  |  |  |  |
| Sex | 0.3946 |  | 1.7906 | 1.8767 |  | 0.0735 | 0.1205 |
| Population | 6.182 |  | 2.8821 | 1.8460 |  | 0.3520 | 0.2958 |
| Days after capture | 0.2846 |  | 1.5353 | 0.0266 |  | 0.3259 | 0.7576 |
| Gecko mass | 141.1476 |  | 0.6174 | 1.1941 |  | 85.6962 | 0.6442 |
| Ectoparasitic mites (unidentified) | 0.4145 |  | 0.9685 | 0.2410 |  | 2.1117 | 0.0964 |
| Pentastomes (*R. indica*) | 2.0597 |  | 1.3314 | 0.0110 |  | 6.8673 | 8.4940 |
| Intestinal tapeworms (*O. javaensis*) | 0.0094 |  | 0.2902 | 0.4050 |  | 0.0204 | 0.0103 |
| Intestinal nematodes A (unidentified pinworms) | 0.9908 |  | 0.0085 | 0.0107 |  | 0.4421 | 1.2207 |
| df (Error) | 51 |  | 51 | 51 |  | 51 | 51 |

**Table D in S2 File**. Summary of explanatory variables (including sex) and their F-values on VCO_2_ for recently active geckos (*n* = 60) (*see* Table A in S2 File). All explanatory variables had 1 degree of freedom (df) and degrees of freedom for each model error are given in the final row.

^a^Variable was log-transformed
